# Gastritis Stages in Monozygotic and Dizygotic Dyspeptic Twins

**DOI:** 10.1155/2020/9510717

**Published:** 2020-07-02

**Authors:** Mindaugas Urba, Jurgita Skieceviciene, Dainius Janciauskas, Laimas Jonaitis, Limas Kupcinskas, Matteo Fassan, Massimo Rugge, Juozas Kupcinskas

**Affiliations:** ^1^Department of Gastroenterology, Institute for Digestive Research, Lithuanian University of Health Sciences, Kaunas LT-50161, Lithuania; ^2^Department of Pathological Anatomy, Lithuanian University of Health Sciences, Kaunas LT 50161, Lithuania; ^3^Department of Medicine (DIMED), Surgical Pathology & Cytopathology Unit, University of Padua, Padua 35121, Italy; ^4^Veneto Cancer Registry, Regional Authority, Padova 35100, Italy

## Abstract

**Background:**

The progression of *Helicobacter pylori*-associated gastritis towards atrophic gastritis is modulated by host-related and environmental factors. Studies that explore the possible involvement of host-related versus environmental factors in the development of gastritis phenotype induced by *H. pylori* are highly needed.

**Aims:**

Our study was aimed at investigating the phenotype of *H. pylori*-associated gastritis in two cohorts of monozygotic and dizygotic twins, using the OLGA/OLGIM gastritis staging system.

**Methods:**

Two cohorts of monozygotic (14 pairs) and dizygotic (15 pairs) dyspeptic twins prospectively underwent endoscopy with biopsy sampling based on Sydney protocol. *H. pylori* status and OLGA/OLGIM stages were assessed and compared.

**Results:**

The mean age of monozygotic and dizygotic twins was 40.4 and 38.6 years, respectively (*p* = 0.623). The overall prevalence of *H. pylori* infection was 51.7%. Among the 14 monozygotic twin pairs, five pairs were *H. pylori*-positive, four were *H. pylori*-negative, and five were *H. pylori*-discordant. Among the 15 dizygotic twin pairs, five pairs were *H. pylori*-positive, five were *H. pylori*-negative, and five were *H. pylori*-discordant. Concordance for antrum atrophy in monozygotic twins was 78.6% (11/14 pairs) and in dizygotic twins 73.3% (11/15 pairs) (*p* = 0.742). Concordance for corpus atrophy in monozygotic versus dizygotic twins was 92.9% (13/14 pairs) and 86.7% (13/15 pairs), respectively (*p* = 0.584). Concordance for antrum intestinal metaplasia (IM) in monozygotic twins was 85.7% (12/14 pairs) and in dizygotic 73.3% (11/15 pairs) (*p* = 0.411). Concordance for corpus IM in monozygotic twins was 85.7% (12/14 pairs) and in dizygotic 86.7% (13/15 pairs) (*p* = 0.941). Among monozygotic and dizygotic subjects, the stage of gastritis was concordant in both *H. pylori*-positive and *H. pylori*-negative subjects.

**Conclusions:**

In conclusion, histological gastric mucosa alterations in monozygotic and dizygotic twins showed high rates of concordance. Furthermore, OLGA/OLGIM gastritis stages were not modulated by the zygosity of the twins.

## 1. Introduction

Gastric carcinogenesis is a multistep process, including a stepwise sequence of phenotypic modifications of the native gastric tissue from healthy gastric mucosa towards atrophic gastritis (AG), intestinal metaplasia (IM), and gastric cancer (GC) [1]. Within this spectrum of lesions, gastric mucosa atrophy is considered the elective “field of cancerization” prone to GC development [1]. *Helicobacter pylori* (*H. pylori*) is by far the most common etiological agent of gastric atrophy, and, all over the world, the prevalence of the *H. pylori* infection is consistently linked with both, AG and GC [1–3].

In some peculiar epidemiological contexts, however, a high prevalence of bacterial infection is associated with a low prevalence of gastric precancerous/cancer lesions, or equivalent rates of bacterial infection are associated with significantly different risk of gastric malignancy [4, 5]. These unexpected findings would support the hypothesis that etiological factors, other than *H. pylori*, may be involved in the modulation of the oncogenetic “cascade.” Among these factors, both host-related (genetic variations, noncoding RNAs, methylation, etc) and/or other environmental factors have been considered [6–14].

To explore the potential host-related factors in the pathogenesis of gastric atrophy, this study compares two populations of dyspeptic monozygotic and dizygotic Lithuanian twins. This is the first study in the field that comprehensively evaluates gastric mucosa alterations in twins.

## 2. Materials and Methods

### 2.1. Study Population

Two cohorts of twins were selected from the Twin Registry Center at Lithuanian University of Health Sciences (years 2016-2018). The twins were interviewed by phone calls, and those that reported clinical symptoms of dyspepsia were referred for upper gastrointestinal endoscopy. Zygosity of twins (mono- versus dizygosity) was confirmed by automated analysis of DNA microsatellite markers. The study included 29 twin pairs (58 subjects) older than 18 years: 14 pairs of monozygotic and 15 pairs (10 same-sex pairs and 5 mixed-sex pairs) of dizygotic twins. None of the considered patients had previously undergone anti-*H. pylori* treatment. All study participants did not use PPIs or antibiotics at least one month prior to inclusion.

### 2.2. Information on Approval of the Local Ethical Committee and Informed Consent

The study protocol was approved by the Ethical Committee of Lithuanian University of Health Sciences (BE-2-10). Informed consent was given for all patients before endoscopy.

### 2.3. Endoscopy and Protocol of Gastric Biopsy Sampling

All the endoscopic procedures were performed by the same trained gastroenterologist at the Department of Gastroenterology of Hospital of Lithuanian University of Health Sciences Kaunas Clinics in order to meet requirements for OLGA and OLGIM histological scoring [14–16]. In all patients, the biopsy protocol included 5 biopsy samples, according to the Sydney recommendations [17]. Biopsy specimens were fixed in formalin (10% solution), embedded in paraffin, and stained with hematoxylin and eosin and Giemsa stain for *H. pylori*. Gastric biopsy specimens were histologically assessed (Sydney score) by a trained pathologist, blinded to any clinical information. In all cases, both the OLGA and OLGIM staging systems were applied, according to the defined criteria [1, 18, 19].

### 2.4. Statistical Analysis

Statistical analysis was performed using IBM SPSS software (v 22.0). Age is presented as mean and median values, while age differences between the groups were evaluated comparing mean values using a *T*-test. The Pearson Chi-squared test to compare sample proportion was applied. The exact Pearson Chi-squared test was used to assess concordance of OLGA and OLGIM stages between monozygotic and dizygotic twins. *p* < 0.05 was considered significant.

## 3. Results

### 3.1. Characteristics of Subjects

Demographics and major clinical findings of twins included within the study are shown in Table 1. The mean and median age (years) of monozygotic and dizygotic twins was 40.3 (39.5) and 38.6 (36), respectively (*p* = 0.623).

### 3.2. *H. pylori* Status in Twins

The overall prevalence of *H. pylori* infection was 51.7%. Concordance rates for *H. pylori* infection prevalence among monozygotic and dizygotic twins was 35.7% and 33.3%, respectively (*p* = 0.891).

Among monozygotic twins, the *H. pylori* status (*Hp*-status) was as the following: five pairs were *Hp*-positive, four pairs were *Hp*-negative, and five pairs were *Hp*-discordant. Among dizygotic twins, five pairs were *Hp*-positive and five pairs were *Hp*-negative, and in five pairs, the *Hp*-status was discordant. Concordant *Hp*-status (both *Hp*-positive and *Hp*-negative) in monozygotic and dizygotic twins was determined in 9/14 (64.3%) and in 10/15 (66.7%) twin pairs, respectively (*p* = 0.893) (Table 1).

### 3.3. Histological Alterations of Gastric Mucosa in Twins

Concordance of topographical extension of atrophic lesions among monozygotic and dizygotic twins is shown in Table 2.

The distribution of monozygotic and dizygotic twins according to OLGA and OLGIM gastritis stages is shown in Table 3. The prevalence of low-risk OLGA and OLGIM stages among mono- and dizygotic twins was 100% and 93.3%, respectively, whereas the prevalence of high-risk stages was 0% and 6.7%, respectively. Concordance according to OLGA and OLGIM stages between monozygotic and dizygotic twins did not reach statistical significance (*p* = 0.097 and *p* = 0.175, respectively).

Tables 4 and 5 show the prevalence of similar or different gastritis stages (grouped in low versus high risk) in monozygotic and dizygotic twins distinguished by their *Hp*-status (*Hp*-positive pair, *Hp*-negative-pair, and *Hp*-discordant pair). Low-risk stages (stages 0-I-II) largely prevailed among both mono- and dizygotic twins (only two cases featured a high-risk stage (stage III, by OLGA and OLGIM). By applying both OLGA and OLGIM staging, no differences emerged in the distribution of twins by stage after performing a comparison of low- versus high-risk stages.

## 4. Discussion

Our study has investigated the phenotype of *H. pylori*-associated gastritis in two cohorts of monozygotic (14 pairs) and dizygotic (15 pairs) twins. The results of our study revealed high concordance rates of gastric mucosal alterations both in monozygotic and dizygotic twins. To our best knowledge, to date, there have been no previous reports, which have assessed the importance of shared genetic influences on susceptibility and phenotype of chronic *H. pylori* gastritis and premalignant gastric alterations on a well-defined twin cohort.

Epidemiological contexts, case-controls, and twins studies have addressed the issue of the possible interaction between different host-related and environmental risk factors in the promotion of gastric precancerous lesions and gastric mucosal atrophy. These factors have been extensively studied among the patients with precancerous gastric lesions and included virulence factors of *H. pylori* strains, environmental factors (smoking, salted food, etc.), or genetic predisposition of the host [20, 21]. To date, however, the exact factors that trigger malignant transformation from normal gastric mucosa towards gastric cancer are not completely understood.

A previous study conducted in Sweden involving monozygotic (36 pairs) and dizygotic (88 pairs) twins reared apart revealed that the concordance rate for *H. pylori* infection was significantly higher among monozygotic twins (82%) than among dizygotic (66%) twins [22]. Slightly higher concordance rates for *H. pylori* infection prevalence among monozygotic (35.7%) versus dizygotic (33.3%) have also been observed in the present study. The difference, however, did not reach statistical significance, probably due to a small number of twin pairs. Based on case-control studies in different geographical regions, which evaluate the prevalence of *H. pylori* infection and the development of gastric atrophy and IM, first-degree relatives of GC patients are associated with an increased risk for GC. A Marcos-Pinto et al. [21] study showed that first-degree relatives of early-onset gastric carcinoma patients have significantly higher prevalence of *H. pylori*, AG, and advanced stages of OLGA. *H. pylori* was present in 82% of cases and in 59% of controls (*p* = 0.001); AG was diagnosed in 70% of cases and in 32% of control individuals, respectively (*p* < 0.001), while OLGA stages III and IV were present in 10% and 9% in both groups, respectively (*p* < 0.001). The results of Rokkas et al. [23] meta-analysis showed similar results: their pooled OR of *H. pylori* infection, AG, and IM between individuals with family history of GC were 1.92-fold (*p* ≤ 0.001), 2.2-fold (*p* = 0.005), and 1.98-fold (*p* ≤ 0.001) higher, respectively, in comparison with controls. These results can be influenced by the exposition of shared environmental factors (alcohol, smoking, salted and smoked food, and hygiene) between family members, circulation of intrafamilial *H. pylori* strains [24–26], and genetic susceptibility [27–29].

Our study showed that there was no difference in concordance rates for chronic *H. pylori* gastritis among monozygotic twins (64.3%, 9/14 pairs) as compared to dizygotic twins 66.7% (10/15 pairs) (*p* = 0.893). This could partly relate to the fact that twins share the same environment in early childhood and genetic predisposition for *H. pylori* acquisition might be less important. Concordance of topographical extension of atrophy and IM was higher among monozygotic twins but did not reach statistical significance. There were no statistical differences in concordance rates according to OLGA and OLGIM stages between monozygotic and dizygotic twins (*p* = 0.097 and 0.175, respectively). It is very important to point out that 11 of 14 monozygotic twin pairs and 10 out of 15 dizygotic twins had absolute concordance for OLGA stages. Similarly, 10 out of 14 monozygotic twin pairs and 10 out of 15 dizygotic twins had absolute concordance for OLGIM stages. These findings suggest that if one twin is identified with high-risk premalignant gastric lesions, the other twin should also be assessed for these alterations.

A relatively small number of the study population represent the main limitation of the present study. The numbers of individual twins within our study group were not very large, and certain effects might have been biased. The design of the study also did not allow us to evaluate as to what extent gastric mucosal alterations in twins are related to the common shared genetic factors per se or shared environmental factors during childhood and later on in lifetime. There might also have been a certain selection bias, because our study included only twins with dyspeptic symptoms.

## 5. Conclusions

In conclusion, histological gastric mucosa alterations in monozygotic and dizygotic twins showed high rates of concordance. Furthermore, OLGA and OLGIM gastritis stages were not modulated by the zygosity of the twins.

## Figures and Tables

**Table 1:** Demographics and *H. pylori* status distinguishing monozygotic versus dizygotic twins.

Twin pairs	Monozygotic *n* (14)			Dizygotic *n* (15)			*p* value
Gender of twins within a pair	MM *n* (3)	FF *n* (11)	MF —	MM *n* (1)	FF *n* (9)	MF *n* (5)	
Mean (median) age		40.3 (39.5)			38.6 (36)		0.623
Concordant *Hp*-positive status		5			5		
Concordant *Hp*-negative status		4			5		0.893
Discordant *Hp*-negative status		5			5		

MM: both twins of male gender; FF: both twins of female gender; MF: twin pair comprised of a male and a female twin.

**Table 2:** Concordance of topographical extension of atrophy and IM in monozygotic and dizygotic twins.

Twin pairs	Monozygotic		Dizygotic		*p* value
	*n* (14)	%	*n* (15)	%	
Concordance according to antrum athrophy	11	78.6%	11	73.3%	0.742
Concordance according to corpus athrophy	13	92.9%	13	86.7%	0.584
Concordance according to antrum IM	12	85.7%	11	73.3%	0.411
Concordance according to corpus IM	12	85.7%	13	86.7%	0.941

IM: intestinal metaplasia.

**Table 3:** Gastritis stage (OLGA and OLGIM systems) in monozygotic and dizygotic twins.

Stage	OLGA staging				*p* value	OLGIM staging				*p* value
	Monozygotic twins		Dizygotic twins			Monozygotic twins		Dizygotic twins		
	*n* (28)	%	*n* (30)	%		*n* (28)	%	*n* (30)	%	
0	18	64.3%	24	80%	0.097	19	67.9%	24	80%	0.175
I	9	32.1%	3	10%		8	28.6%	3	10%	
II	1	3.6%	1	3.3%		1	3.6%	1	3.3%	
III	—	—	2	6.7%		—	—	2	6.7%	
IV	—	—	—	—		—	—	—	—	

OLGA: Operative Link on Gastritis Assessment; OLGIM: Operative Link on Gastritis/Intestinal-Metaplasia Assessment.

**Table 4:** Gastritis OLGA-stage according to the *H. pylori* status: twins are distinguished according to zygosity.

Group of stages	Twin pair *Hp*-positive (10 pairs)		Twin pair *Hp*-negative (9 pairs)		Twin pair *Hp*-discordant (10 pairs)	
	Monozygotic	Dizygotic	Monozygotic	Dizygotic	Monozygotic	Dizygotic
Similar low-risk stage (0-I-II)	5	4	4	5	5	4
Similar high-risk stage (III-IV)	—	—	—	—	—	—
Discordant stage (0-I-II versus III-IV)	—	1	—	—	—	1

OLGA: Operative Link on Gastritis Assessment; *Hp*: *H. pylori*.

**Table 5:** Gastritis OLGIM-stage according to the *H. pylori* status distinguished by zygosity.

Group of stages	Twin pair *Hp*-positive (10 pairs)		Twin pair *Hp*-negative (9 pairs)		Twin pair *Hp*-discordant (10 pairs)	
	Monozygotic	Dizygotic	Monozygotic	Dizygotic	Monozygotic	Dizygotic
Similar low-risk stage (0-I-II)	5	4	4	5	5	4
Similar high-risk stage (III-IV)	—	—	—	—	—	—
Discordant stage (0-I-II versus III-IV)	—	1	—	—	—	1

OLGIM: Operative Link on Gastritis/Intestinal-Metaplasia Assessment; *Hp*: *H. pylori*.

## Data Availability

The authors can make research data available on request after institutional review board approval through contacting the corresponding author by juozas.kupcinskas@lsmuni.lt
